# The Financial Strength of Our Voluntary Charities

**Published:** 1895-01-05

**Authors:** Henry C. Burdett


### THE INSTITUTIONAL WORKSHOP.

### THE FINANCIAL STRENGTH OF OUR VOLUNTARY CHARITIES.

By HENRY C. BURDETT.

[The *Times*, in a leading article, having argued that charity was *in extremis*, a reply to this contention appeared in the *Times* on the 27th ult.]

"Circumstances have brought it about that each year I find myself more and more in the position of Chancellor of the Exchequer for the British charities, as the reports and accounts of nearly all the charities are sent to me by the authorities of these institutions. The figures are carefully examined, collated, and classified, and a general statement prepared of the financial position they reveal. These figures are published each year in the 'Hospital and Charities Annual,' and it is hoped that they will be studied much more closely in the future than they have been in the past. They show by demonstration that the opinions expressed in the article on 'Charity *in extremis*' are not borne out by the facts. Taking the great hospitals, we must first deal with the capital side of the account. The article refers to the case of Guy's Hospital, and states that its annual income has now fallen off by £20,000, owing to the fall in the rents of the estates of the hospital. With the exception of the Royal hospitals, the invested property of our hospitals does not consist to any material extent of land. It is mostly sunk in high-class dividend-paying investments. The case of Guy's Hospital is exceptional, and had the same wisdom been displayed in the management of its affairs years ago as was shown by those responsible for St. Bartholomew's Hospital, there can be no doubt that this fall in the income would have been enormously reduced or entirely prevented. One hundred and fifty-nine English, Scotch, and Irish general and special hospitals have an income of upwards of £380,000 per annum from dividends and invested property, that is to say, that upwards of 38 per cent. of their total income is derived from this source. This income is not altogether a source of strength, because there is a tendency in the case of institutions which do not depend mainly upon the income which they derive each year direct from the public to allow the administration to be less efficient than it might and ought to be. I am confident after thirty years' experience that it is not desirable as a general rule for a voluntary charity to have invested property to a larger amount than the sum represented by from five to ten years of its average annual expenditure. It follows that there ought to be a limit to the reserve fund which the managers of a charity build up, and in the lean years and periods of depression, when values shrink and the people as a whole experience less than an average prosperity, that resort should be had to the reserve fund to make up any deficiency in income.

### HOSPITALS IN 1885, 1892, AND 1893.

"The financial position of the 159 special and general hospitals during the year 1892 was as follows: Taking Great Britain and Ireland as a whole, the income exceeded the expenditure not only in the case of the general hospitals, with and without medical schools, but in every group of special hospitals from consumption to miscellaneous. Indeed, in several instances the balance in favour of income was very large, being upwards of £25,000 in the case of general hospitals with medical schools, and upwards of £71,000 in the case of general hospitals without medical schools. The special hospitals received as a whole an income of £331,236, and expended £251,855, leaving a balance in their favour for 1892 of something like £80,000, or 33 per cent. of the total expenditure for the year. The revenue during 1892 of every class of metropolitan hospitals, with the exception of that for women and children, exceeded their expenditure.

"Lest it should be said that 1892 was an exceptional year, I think it necessary to give the following table, which contains the actual figures, prepared upon an identical basis, and published by the council of the Metropolitan Hospital Sunday Fund, for the years 1885 and 1893:—

### METROPOLITAN GENERAL AND SPECIAL HOSPITALS.

Table showing the increase in the work done, the income received, and the expenditure of the metropolitan hospitals during the years 1885 and 1893 compared.

| Year. | Number of Beds. | In-patients. | Out-patients. | Total Expenditure. | Total Income. |
|---|---|---|---|---|---|
| 1893 ... | 8,212 | 83,341 | 1,308,813 | £689,863 | £516,375 |
| 1885 ... | 6,765 | 58,964 | 941,511 | £491,634 | £451,299 |
| Increase in 1893 over 1885 ... | 1,447 | 24,377 | 367,302 | £198,229 | £65,076 |
| Increase per cent. ... | 21·389 | 41·342 | 39,011 | 40·32 | 14·419 |

"The above table shows that there has been an increase under every head in the year 1893, as compared with the year 1885, in the amount of work done to the extent of 21 1-3 per cent. in the number of beds available for patients, of 41½ per cent. in the number of in-patients, and of 39 per cent. in the number of out-patients admitted to treatment. The total income of the participating charities in 1893, as compared with 1885, shows an increase of rather less than 14½ per cent., whereas their total expenditure in 1893, compared with that of 1885, shows an increase of 40⅓ per cent. These figures are irrespective of the large sum received in legacies by the metropolitan hospitals, which ranges from a sum of £300,000 in a good year to £150,000 in a bad year like 1893, when there was a net deficiency between total income, including legacies, and total expenditure of about £22,000. Legacies are the secret which explains how the voluntary hospitals are able to live, and not only to live, but to extend their work year by year, notwithstanding that most years show a deficiency between ordinary income and ordinary expenditure. In the year 1892, for example, the legacies to London hospitals amounted to upwards of £276,000. The foregoing figures prove that it is untrue of the great hospitals as a whole that 'their incomes have diminished, are diminishing, and threaten to diminish more.' The facts, in truth, prove just the contrary.

The General Charities, 1868 to 1893.

"Let us take next the general charities. London contains a greater number of charities of all kinds than any other city in the world, and the combined revenue of these charities is so great as to stagger the uninitiated when brought face to face with the total for the first time. The income of the greater charities which have their headquarters in London amounts to upwards of £7,000,000 sterling per annum, a sum which exceeds the total annual revenue of all but three of the British colonies—*i.e.*, New South Wales, Victoria, and Canada—at the present time. It would be wearisome to deal with the figures in detail. Such an attempt is fortunately not necessary. The attitude of the giving public during the last quarter of a century and the volume of charity which flows into the coffers of the general charities can be fairly realised by quoting the experience of Dr. Barnardo. Dr. Barnardo's methods, so far as the publication of his accounts is concerned, seeing that these accounts as published are mere summaries and totals, does not commend itself to the judgment of business men. Still, the work he does is good work and the volume of that work is immense. It includes general, ecclesiastic, medical, and temperance work, orphanages, convalescent institutions, a Babies' Castle, refuges, children's agencies, emigration missions, and boarding-out—that is to say, the whole field of general charity. Is it true that the incomes of his various institutions have diminished, are diminishing, and threaten to diminish more? Not at all. On the contrary, whereas the income for the year 1868 was but £215, in 1878 it had increased to £32,124, in 1888 to £98,709, and in 1893 to £134,054. Nor is this all, for the report shows not only the amount received, but the number of givers from whom it came. Thus we find that in 1889 the number of donations was 75,748. They fell to 68,368 in 1890, although the total income increased. In 1891 the number of donations was 76,590, and in 1893 82,709. These figures are conclusive and eloquent. It will, therefore, be seen that the opinions expressed in regard to our great hospitals and general charities are not supported by the facts, and that charity so far as they are concerned is not *in extremis*, but very much alive and on the increase. I may further add that the total receipts of the Metropolitan Hospital Sunday Fund this year are greater than those of any other except one (1891) since the fund was established 22 years ago.

The Churches and Local Charities.

"The position of the churches and the local charities is a more difficult matter to speak on. As one who has given a good deal of attention to the subject, I have reason to believe that the figures in regard to them, taken as a whole, could they be produced, would show a similar progress to those just recorded in regard to the two other groups. This view is confirmed by the returns issued by the Church of England in the course of the year, which prove to demonstration that church building, church extension, and the material works of the Church were never greater or in a more active state of progress than they are to-day. At the present time in London a movement is quietly but hopefully extending in the direction of the establishment of inter-denominational committees who take charge of areas of population which can be readily worked by such an organisation. As the movement grows, overlapping will be prevented, the inhabitants of each locality will learn the needs, and will, I am confident, provide the necessary funds to meet the requirements of their own poor. In this way the rectors of the various parishes will be able to know the names of the local charities meriting support, and the multiplication of appeals can be put an end to once and for all.

Causes of Pessimism.

"Having thus brought out the facts, let us proceed to examine what is the explanation of the growth of opinions so ill-founded as those which generally prevail in regard to the volume of charity in this country in these days. It cannot be questioned that the historian of present-day events must record that the people of the period suffered from a pessimism so intense as to be almost past praying for. Most people are so occupied in dwelling on the miseries of the past and the hopeless prospects which the future offers, that they have neither time nor inclination to live in the present or to be thankful for its wonderful privileges and blessings. That is the first explanation. Next we have to reckon with the policy favoured by the majority, I am afraid, of those who have to raise the incomes of our charities at the present time. The prevailing belief seems to be that to be successful an appeal must make out the most terrible case of financial distress and be drawn from the point of view of proving that nothing but bankruptcy awaits the particular charity unless a definite and large sum of money is subscribed within a fortnight. I know one charity of repute which carries this system to the verge of dishonesty; and it would not be difficult to show that appeals of this character, when compared with the published statement of accounts, are often wide of the facts. The circulation of such appeals produces a feeling of despair in the minds of the uninitiated, and so public opinion is forced into an attitude of believing that our charities, and that English charity too, is *in extremis*. The late Mr. Spurgeon once told me, and my experience supports his view, that if you have a large sum of money to raise for a worthy object, incisive truthfulness and the assurance that, if the money is given, the particular charity shall be kept free from debt, is an appeal which invariably meets with a liberal and ready response. The opposite policy is unworthy of men of business, to say nothing of Christians and philanthropists, and we have had a good deal too much of it of late in this country in regard to charitable appeals of all kinds. I would urge the managers of our charities to exhibit more robustness and fibre, and more faith, not only in the love of good works on the part of their countrymen, but in the soundness of the particular charity which they have set themselves to control. Again, there is a want of originality about the appeals, as well as in the methods used to raise money. I am confident that in the case of every charity which is conducted upon business lines, where the managers treat the whole matter of raising income and conducting its affairs upon the soundest business principles, generous support is always forthcoming from the public. It is too much the practice to go over and over the same ground—*i.e.*, to appeal to the same people. There must be a fallow time in charity as well as in agriculture, and those who do not realise this do not deserve to meet with a liberal response to their demands.

REMEDIES SUGGESTED.

"We want more young men upon the committees of our charities in London at the present time. The history of the Poplar Hospital during the last few years is a striking proof of the truth of this contention. The activity of the members of a provincial committee, compared with the irregular attendances and lukewarm interest of some members of several metropolitan committees is most instructive. I should like to hear from the chairman of any committee who would welcome new blood, and also to have the names of any of the younger men who would like to take part in the management of a hospital or other charitable institution, so that supply and demand may be adjusted and a better state of things may ensue.

"Finally, it is necessary to recognise that the last few years have been exceptional years for most people. Not only has taxation increased, may I say wisely increased as to its incidence at any rate, but there has been a steady and almost continuous fall in values. Hence many of the most liberal givers, as they have realised year by year the shrinkage which has taken place in their securities, have felt, as prudent men, that they must diminish for the time being the amount of their gifts, because they were not only suffering from a reduction of income themselves, but that capital values were also affected so seriously as to demand the utmost caution. No doubt most charities have felt the effects of this prolonged depression to an increasing extent, but, on the whole, the diminished resources of our charities are by no means serious, and they could and ought to be met out of the reserve fund, which, in the case of some institutions is, in my judgment, unnecessarily large. A sharp controversy has taken place during the year at Glasgow as to the relative financial strength of some of the large hospitals. It was amusing to notice that the institution which appeared to exhibit the greatest deficiency was the one which was building up the largest reserve fund. This system of saving on the part of charities may be carried a great deal too far. It only tends in the direction of too economical expenditure, and often prevents wise developments and reforms which make for increased efficiency.

BRITISH CHARITY IN A HEALTHY AND PROGRESSIVE CONDITION.

"Looking at the case all round, I am confident that English charity is not *in extremis*, but on the contrary it never was in a healthier or more progressive condition than at the present time. There is no list, official or otherwise, of bankrupt charities, because no well-managed charity is ever bankrupt, and in the whole of my thirty years' experience I have never known one single instance of the kind. It follows from the facts and figures here given that those who take the keenest interest in our charities may thank God and take courage. A little readjustment of system here, a change in officers there, and new blood on the committee of this or that institution will speedily drive out the spirit of pessimism which prevails and substitute for it a spirit of thankfulness, of hope, and success. This new spirit would speedily effect a revolution. With its advent, opinions not founded upon facts, which have tended to cripple the energies of some of the best men and women in the field of philanthropy, will disperse like fog before the rising sun. Under such influences truth will prevail, and all classes of people will soon realise, not only that 'the great fortresses of science and benevolence erected where suffering most abides are the real glory and abiding distinction of our civilisation,' but that they will continue to abide and flourish so long as England is a nation."

